# Clinical findings and predictive factors for positive anti-interferon-γ autoantibodies in patients suffering from a non-tuberculosis mycobacteria or *Talaromyces marneffei* infection: a multicenter prospective cohort study

**DOI:** 10.1038/s41598-022-13160-x

**Published:** 2022-05-31

**Authors:** Ye Qiu, Mengxin Tang, Wen Zeng, Xin Feng, Mianluan Pan, Wei Li, Jianquan Zhang

**Affiliations:** 1 Department of Respiratory and Critical Medicine, The Eighth Affiliated Hospital, Sun Yat-Sen University, Shenzhen, 518000 Guangdong China; 2 State Key Laboratory of Respiratory Disease, National Clinical Research Center for Respiratory Disease, The First Affiliated Hospital of Guangzhou Medical University, Guangzhou, 510120 China; 3 Department of Respiratory and Critical Medicine, The First Affiliated Hospital of Guangxi Medical University, Nanning, 530021 Guangxi China; 4 Department of Nephrology, The Second Affiliated Hospital of Guangxi Medical University, Nanning, 530021 Guangxi China

**Keywords:** Immunology, Microbiology

## Abstract

We investigated the clinical features and screened for predictive factors of anti-interferon-γ autoantibody (AIGA) positivity. We enrolled 63 AIGA-positive (group 1) and 29 AIGA-negative (group 2) HIV-negative patients. White blood cell (WBC) and neutrophil counts, erythrocyte sedimentation rate (ESR), and C-reactive protein (CRP), globulin, immunoglobulin (Ig) G, and IgM levels were higher, whereas CD4^+^T cell count and hemoglobin level were lower in group 1 than in group 2. Co-infections, multiple infections, and disseminated infections were significantly higher in group 1 than in group 2. Prognosis was worse in group 1 than in group 2, especially for relapse and persistent infections. The number of infecting pathogens and sites involved; WBC and neutrophil counts; globulin, IgG, IgM, and CRP levels; and ESR were significantly positively correlated with AIGA titers; however, CD4^+^T cell count was significantly negatively correlated with AIGA titers. Therefore, IgG, globulin, and CRP levels; CD4^+^T cell and WBC counts; the number of infecting pathogens and sites involved; and ESR were considered potential predictors for AIGA positivity. For HIV-negative hosts with double or multiple opportunistic, disseminated infections and high serum IgG and globulin levels, low CD4^+^T cell count, and an increase in inflammatory marker levels, positive AIGA-associated immunodeficiency should be considered.

## Introduction

Adult-onset immunodeficiency syndrome (AOID), caused by anti-interferon-γ autoantibody (AIGA), has been strongly associated with intracellular opportunistic infections in human immunodeficiency virus (HIV)-negative adults^1–6^. In patients with positive AIGAs, several molecular mechanisms underlie interferon (IFN)-γ dysfunction, considered to primarily inhibit signal transducer and activator of transcription 1 (STAT1) phosphorylation and interleukin-12 production, resulting in severe dysfunction of the Th1 response^1,7^. Patients with positive AIGAs manifest clinically severe mycobacterial, *Talaromyces marneffei* (TM), and salmonella infections, with double or multiple infections^1–6^.

Since 2012, an increasing number of patients have been diagnosed with AIGA, suggesting that AIGA production was previously underestimated. To date, more than 800 patients have been diagnosed with AIGAs^7^. The diagnosis of AIGA disease requires the following two steps: 1) the detection of the AIGA titer by enzyme-linked immunosorbent assay (ELISA), particle-based assay, flow cytometry analysis, or western blotting; and 2) an assessment of the neutralizing activity of these AIGAs: STAT-1 phosphorylation or human leukocyte antigen (HLA)-DR expression on IFN-γ-responsive cell assays are the most widely used methods for assessing the neutralization potential of AIGA^1,7,8^. AOID has always been misdiagnosed due to the lack of clinical knowledge about it. However, the AIGA titer is closely related to poor infection prognosis, especially in relapsed and refractory infections^6^. Thus, the timely detection of AIGA and diagnosis of AOID is essential to improve the prognosis of infected patients. However, these methods of diagnosing positive AIGAs are time-consuming, require expensive instruments, and are used only for scientific research rather than a routine clinical test.

Therefore, in this study, we (1) compared AIGA-positive and AIGA-negative patients to elucidate the clinical characteristics of AIOD and (2) screened existing clinical routine indicators to identify potential predictors of AIGAs for timely identification of AIGA positivity to evaluate host immunity and improve prognosis.

## Results

### Baseline characteristics and AIGA titers

Based on the 99th percentile of the AIGA titers in 103 healthy volunteers, the cutoff for positivity was 6402.28 ng/ml. During the study, 63 AIGA-positive cases (group 1) and 29 AIGA-negative cases (group 2) with TM and/or nontuberculosis mycobacteria (NTM) infection were enrolled. All 93 participants were HIV-negative. Sex distribution, age, and body mass index (BMI) did not differ significantly between the groups (Table 1). The AIGA titers in group 1 (median 32,343.8 ng/ml with interquartile range 19,712.8–58,117.3 ng/ml) were significantly higher than those in group 2 (median 3452.9 ng/ml with interquartile range 1985.7–3983.2 ng/ml) (*P* < 0.001).

**Table 1:** Baseline demographics and clinical characteristics of the 92 participants.

Variable	Group 1 (n = 63)	Group 2 (n = 29)	*P*
Age (year)	53 (45–63)	57 (50–63)	0.32
Sex, female n (%)	29 (45.3)	15(51.7)	0.91
BMI (kg/m^2^)	19.5 (16.9–21.9)	20.3 (17.7–21.8)	0.53
Duration of follow up (m)	18.5 (13–38.7)	14.0 (10–33)	0.37
No. of infecting pathogens*	2 (1–6)	1 (1–1)	**0.00**
Coinfected ≥ 2 pathogens n (%^) #^	29 (45.3)	0(0)	–
No. of involved sites	4 (3–6)	1(1–3)	**0.00**
AIGA positive n (%)	64 (100.0)	0 (0)	–
AIGA titers (ng/ml)	32,343.8 (19,712.8–58,117.3)	3452.9 (1985.7–3983.2)	**0.00**
WBC × 10^9^cells/L	18.8 (10.7–24.8)	8.7 (5.9–19.7)	**0.01**
N × 10^9^ cells/L	15.6 (7.7–20.6)	6.9 (3.96–17.0)	**0.03**
L × 10^9^ cells/L	1.4 (1.0–1.9)	0.9(0.3–1.2)	**0.01**
HGB g/L	82.8 (70.8–96.0)	114.2 (70.8–122.2)	**0.03**
ESR mm/h	97.0 (61.5–113.0)	25.0 (10.0–84.0)	**0.00**
CRP mg/L	138.2 (92.5–192.0)	17.8 (10.0–138.3)	**0.01**
CD4^+^T cell cells/µL	484 (365–654)	890 (696–1117)	**0.00**
CD8^+^T cell cells/µL	455 (367–792)	422 (346,822)	0.34
CD3^+^T cell cells/µL	903 (672–1301)	1246 (897–1550)	0.42
IgG g/L	26.6 (20.7–34.7)	12.1 (9.6–16.0)	**0.00**
IgA g/L	3.0 (2.2–4.1)	2.3(2.1–2.7)	0.14
IgM g/L	1.5 (1.2–2.4)	0.7 (0.4–1.2)	**0.00**
Globulin g/L	41.6 (36.1–55.4)	24.8(20.7–28.5)	**0.00**
**Prognosis and outcomes**			**0.04**
Cured	15 (23.4)	15 (51.7)	
Persistent infection	19 (29.7)	5 (17.2)	
Relapse infection	21 (32.8)	6 (20.7)	
Death	9 (14.1)	3 (10.3)	

Data are expressed as median ± interquartile range. Fisher’s exact test and Kruskal–Wallis H test were used to determine statistical significance among the groups. *P* < 0.05. Data were collected under sterile conditions before the patient received antimicrobial therapy treatment and during the active stage of the infection. Group 1 = AIGA-positive patients, Group 2 = AIGA-negative patients.

*The number of infecting pathogens is expressed as median (range minimum to maximum).

^#^Among these, 29 patients showed co-infection or multiple infections, including 4 with TM and *Salmonella* co-infection, 2 with TM and *Burkholderia* co-infection, 2 with TM and *Klebsiella pneumoniae* co-infection, 1 with TM and *Staphylococcus aureus* co-infection, and 20 with TM and NTM co-infection. Among the 20 patients with TM and NTM co-infection, 13 were infected with more than three pathogens**.** Among these 13 patients, besides TM and NTM, 2 patients were infected with *Staphylococcus aureus*, 3 with *Aspergillus*, 3 with *Salmonella*, 3 with *Burkholde*ria, 1 with *Candida albicans*, 1 with *Klebsiella pneumoniae*, 1 with *Providencia rettgeri*, 1 with *Citrobacter*, and 1 with Epstein-Barr virus. *BMI* body mass index; *AIGA* anti-IFN-γ autoantibody; *ND* not done; *WBC* white blood cell; *N* neutrophil count; *L* lymphocyte count; *HGB* hemoglobin; *ESR* erythrocyte sedimentation rate; *CRP* C-reactive protein; *Ig* immunoglobulin. Normal range: IgG: 8–18 g/L, IgA: 2.01–2.69 g/L, IgM: 0.84–1.32 g/L, CD4^+^T cell: 410–1590 cells/µL, CD8^+^T cell: 190–1140 cells/µL, CD3^+^T cell: 690–2540 cells/µL.

Significant values are in [bold].

### Comparison of clinical features and outcomes between the AIGA-positive and AIGA-negative groups

A comparison of the biomarkers and clinical parameters between the groups (Table 1) revealed that the AIGA titer, white blood cell (WBC) count, neutrophil (N) count, erythrocyte sedimentation rate (ESR), and C-reactive protein (CRP) level in group 1 were higher than those in group 2 (*P* < 0.00). The hemoglobin (HGB) level in group 1 was lower than that in group 2 (*P* < 0.01). Furthermore, the globulin, immunoglobulin (Ig) G, and IgM levels were higher in group 1 than in group 2 (*P* < 0.05). In group 1, there were 29 (45.3%) patients with co-infections or multiple infections, with the co-infection rate being significantly higher than that in group 2 (*P* < 0.001). Besides, the number of sites involved in group 1 was significantly higher than that in group 2 (*P* < 0.001) (4 sites vs. 1 site, respectively) (Table 1).

In group 1, the median CD4^+^ T lymphocyte count was 484 (with interquartile range 365–654) cells/µL, lower than the normal range. In addition, the CD4^+^ T lymphocyte count in group 1 was lower than that in group 2 (*P* < 0.01). The CD4^+^ T lymphocyte count of nine patients in group 1 was below the normal level (CD4^+^T cells < 410 cells/µL), and all nine patients had disseminated infections, persistent infections, and co-infections.

The prognosis and outcomes of patients in group 1 were worse than those of patients in group 2, especially in the case of persistent and relapse infections (*P* < 0.001) (Table 1). Nineteen (29.7%) patients had a persistent infection, 21 (32.8%) had a relapse infection, 9 (14.1%) died, and 15 (23.4%) were cured in group 1. In contrast, 5 (17.2%) patients had a persistent infection, 6 (20.7%) had a relapse infection, 3 (10.3%) died, and 15 (51.7%) were cured in group 2.

### Pearson correlation and univariate logistic regression analysis for predictive factors of AIGA positivity

**Figure 1:**
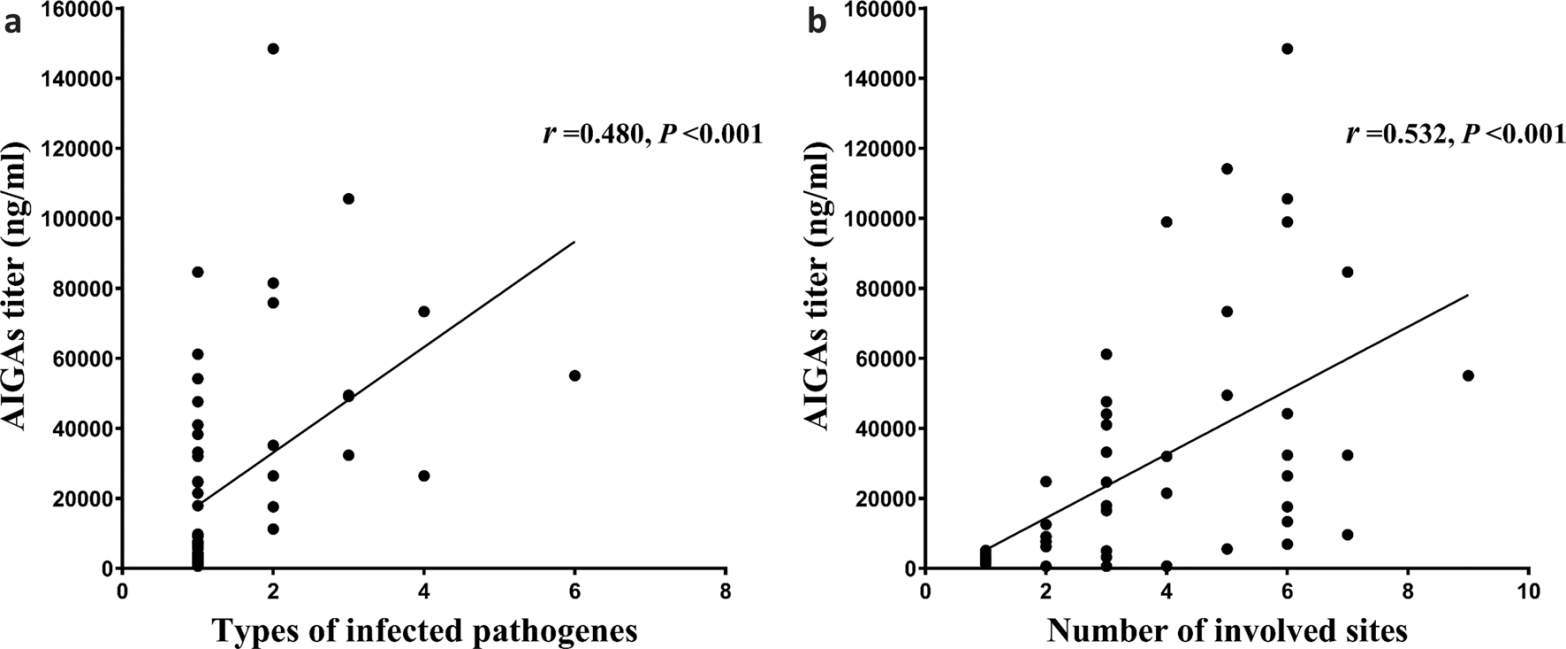
Pearson correlation analysis among anti-IFN-γ autoantibodies, the number of infectious pathogens, and involved sites. *P* < 0.05.

**Figure 2:**
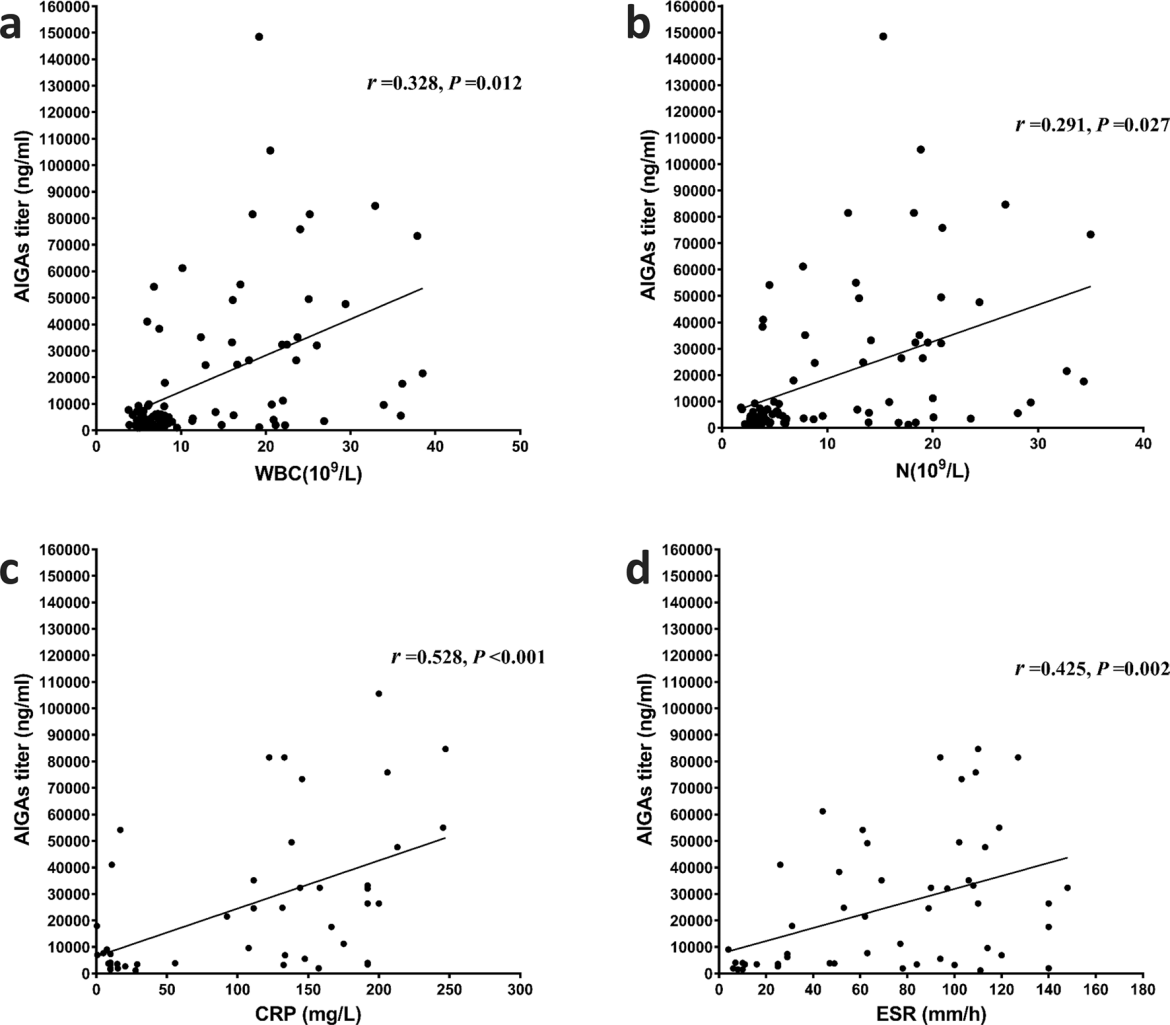
Pearson correlation analysis between anti-IFN-γ autoantibodies and inflammatory markers. WBC (**a**), N (**b**), CRP (**c**), and ESR (**d**). *P* < 0.05. *AIGA* anti-IFN-γ autoantibody; *WBC* white blood cell; *N* neutrophil count; *ESR* erythrocyte sedimentation rate; *CRP* C-reactive protein.

**Figure 3:**
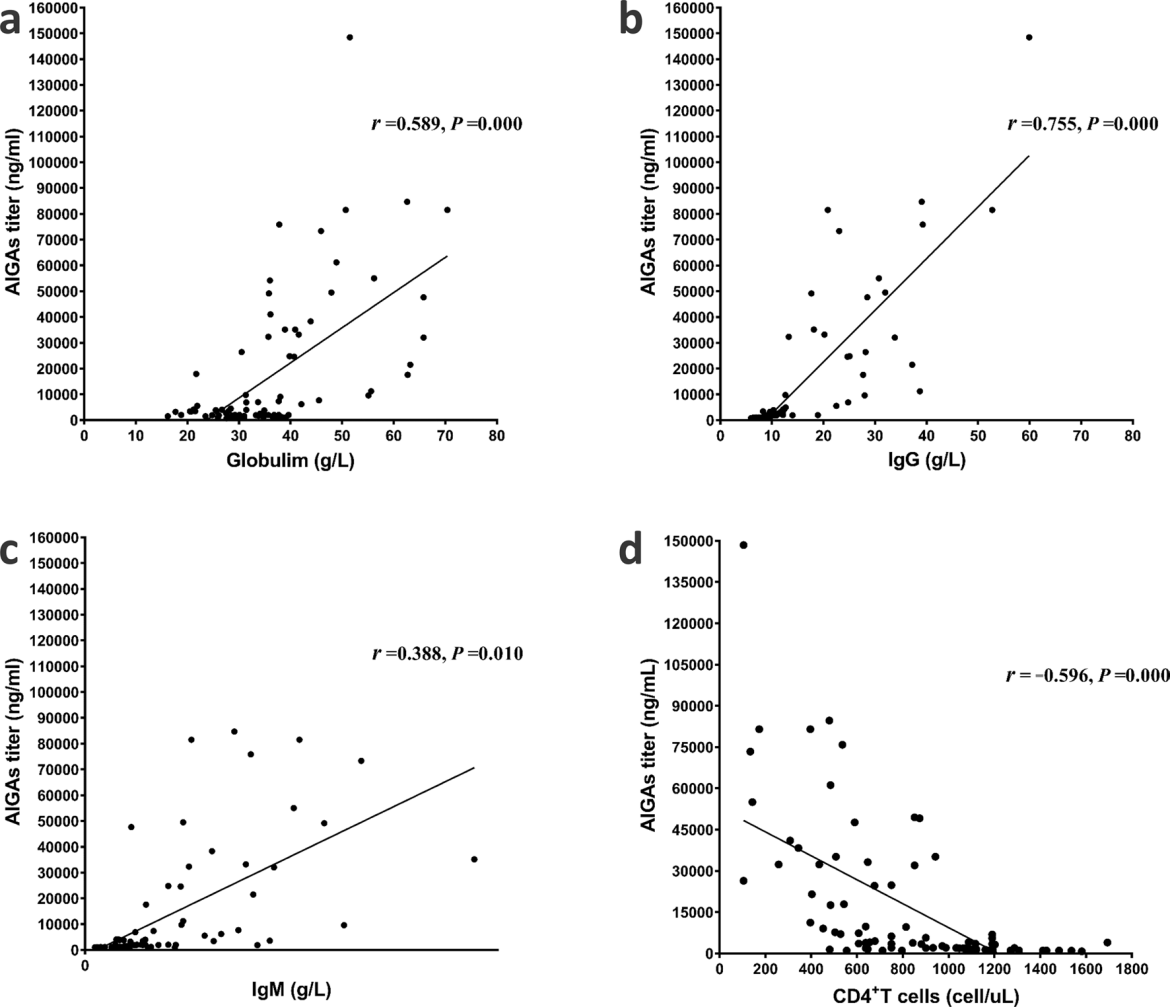
Pearson correlation analysis between anti-IFN-γ autoantibodies and immune indexes. Globulin (**a**), IgG (**b**), IgM (**c**), and CD4 + T cells (**d**). *P* < 0.05. *AIGA* anti-IFN-γ autoantibody, *Ig* immunoglobulin.

Pearson correlation analysis with a two-tailed test was used to correlate between AIGAs and clinical indices. The number of different types of infecting pathogens (*P* = 0.000, *r* = 0.480) and number of sites involved (*P* = 0.000, *r* = 0.532) (Fig. 1); WBC (*P* = 0.012, *r* = 0.328) and neutrophil counts (*P* = 0.027, *r* = 0.291); ESR (*P* = 0.002, *r* = 0.425); and CRP (*P* = 0.000, *r* = 0.528) (Fig. 2), globulin (*P* = 0.000, *r* = 0.589), IgG (*P* = 0.000, *r* = 0.755), and IgM (*P* = 0.010, r = 0.388) (Fig. 3) levels significantly positively correlated with AIGA titers. The CD4^+^T cell count significantly negatively correlated with AIGA titers (*P* = 0.000, *r* =  − 0.596) (Fig. 3). The univariate logistic regression analysis showed that the CD4^+^T cell; the number of types of infecting pathogens and sites involved; IgG, globulin, and CRP levels of patients are potential predictors for AIGA positivity (Table 2).

**Table 2:** Results of the univariate analysis for AIGA positivity (n = 92).

Variable	*P*	*r*	R^2^
Types of infecting pathogens	0.000	15,023.178	**0.230**
No. of involved sites	0.000	8598.168	**0.310**
Underlying disease	0.680	3436.103	0.003
WBC × 10^9^cells/L	0.012	1048.2	0.107
N × 10^9^cells/L	0.027	1019.579	0.085
HGB g/L	0.051	− 275.4	0.066
CD4^+^ T cell cells/µL	0.000	− 52.544	**0.355**
IgG g/L	0.000	2017.025	**0.570**
IgM g/L	0.010	13,142.694	0.150
GLO g/L	0.000	1250.074	**0.347**
ESR mm/h	0.002	293.641	0.180
CRP mg/L	0.000	211.043	**0.279**

Data were collected under sterile conditions before the patient received antimicrobial therapy and during the active stage of the infection. *AIGA* anti-IFN-γ autoantibodies; *WBC* white blood cell; *N* neutrophil count; *ESR* erythrocyte sedimentation rate; *CRP* C-reactive protein; *Ig* immunoglobulin. *r* regression coefficient.

Significant values are in [bold].

## Discussion

Until now, the diagnostic criteria for Adult-onset immunodeficiency syndrome (AOID) have been unavailable, the clinical knowledge and attention about AOID have been limited, and routine clinical tests for detecting AIGAs have been lacking. These resulted in the misdiagnosis of AOID . Therefore, finding potential clinical predictors of AIGA positivity from existing clinical indicators is important for timely detection and diagnosis of AIGAs to evaluate host immunity and improve prognosis. In this study, we determined significant predictors for AIGAs that could play a role in controlling the disease. This study provides the first evidence that high globulin and IgG levels, low CD4^+^T levels, double or multiple infections, and disseminated infections could be potentially effective predictive factors of AIGA positivity in HIV-negative patients with TM and/or NTM infections.

In this study, the inflammatory markers for leukocyte count, CRP level, and ESR in AIGA-positive patients were significantly higher than those in AIGA-negative patients. A notable bone marrow response prone to anemia and leukocytosis development in AIGA-positive patients was also observed. In addition, most patients who were AIGA-positive had systemic dissemination and double or multiple infections frequently. These markers indicate more exuberant dissemination and multiple infections in patients with AIGA positivity.

We found that globulin, IgG, and IgM levels in AIGA-positive patients were higher than those in AIGA-negative cases. Furthermore, the AIGA titer was significantly positively correlated with IgG and serum globulin levels in patients. The globulin level was high because the essence of AIGAs is immunoglobulins. After total immunoglobulin subclass evaluation and functional verification, including AIGA IgG, IgM, and IgA subclasses, Browne et al. found that the isotypes and subtypes of AIGAs appear to be heterogeneous, and IgG1 and IgG4 were the most frequent subtypes in the population^1,6,8^. Moreover, AIGA IgG can inhibit IFN-γ-dependent STAT1 phosphorylation, especially IgG4^8^. However, AIGA IgA and IgM have binding activity; they do not neutralize inhibitory interferon-γ-dependent STAT1 phosphorylation^1,8^. These issues indicated that IgG-AIGA is the major subtype of AIGAs and neutralizing component to prevent interferon-γ-induced STAT1 phosphorylation. Therefore, the high IgG level in peripheral blood can be used as an effective predictive factor in AIGA-positive patients, and the level of IgG can be used to evaluate the intensity of immunosuppression in serum.

The number of infecting pathogens is significantly positively correlated with AIGA titers, which may be associated with pathogens expressing the Noc2 protein. Lin CH et al. found that pathogenic AIGAs may be produced from cross-reactivity between *Aspergillus spp.* and *M. intracellulare* ribosomal assembly protein Noc2, especially Noc2 in Aspergillus spp. Furthermore, these autoantibodies target a major epitope (P_123–135_ AAKTGKRKRSQML) in the C-terminal region of IFN-γ^5^. To investigate whether the Noc2 protein in TM has a similarly form of molecular mimicry that could trigger the development of AIGAs, we searched in NCBI BLAST for sequences displaying homology to P_123–135_, which sequence was unique to IFN-γ in humans. Surprisingly, the P_123–135_ epitope had 80%-positive homology to amino acids 98–107 (AKTGKRKRID) of the ribosome assembly protein Noc2 of TM. At the same time, the two sequences are similar in spatial structure. This region in Noc2 is highly conserved across all of the species in the databases (UniProt). Therefore, when infected with NTM, TM, *Aspergillus*, and other pathogens with Noc2 sequences, cross-reactivity with Noc2 might occur, stimulating the production of AIGAs, leading to further impaired immunity. In the present study, the AIGA-positive titer of patients with co-infection and multiple infections was significantly higher than that in AIGA-negative patients. Moreover, the AIGA titer was significantly positively correlated with the number of pathogens that infected the patients. These findings are consistent with those of previous studies and our hypothesis. However, further investigation is required to determine whether the AIGA titer positively correlates with the number of pathogens or the Noc2 level.

As the clinical manifestations and recurrent opportunistic infection of AOID are similar to those if AIDS, AOID caused by AIGAs has previously been defined as an “AIDS-like syndrome”^9,10^. However, the mechanisms of immune deficiency between them are completely different. Previously, studies have found that persistent exposure to antigen continuously stimulates T lymphocytes leading to prolonged inflammation. During chronic infections, memory T lymphocytes enter an entirely different differentiation program that ends in T cell exhaustion^11,12^. Here, the CD4^+^ T cell count was lower in AIGA-positive patients than in AIGA-negative patients. Meanwhile, there was a significant negative correlation between the AIGA titer and CD4^+^ T cell count. These may be related to the following reasons. First, post-infection immunosuppression. The CD4^+^ T cell count was selected during the active stage of infection. Co-infection and multiple, persistent, and disseminated infections were more common in AIGA-positive patients than in AIGA-negative patients. Second, because of its ability to regulate various protective functions and sustain the activity of both CD4^+^ and CD8^+^T cells, IFN-γ is essential for fighting infections^13^. Third, AIGAs can underlie IFN-γ dysfunction by inhibiting STAT1 phosphorylation, interleukin-12 production, and severe dysfunction of the Th1 response^1,7,14^ and affect the differentiation of CD4^+^ T cell subsets, leading to a decrease in Th1 cell differentiation and proliferation.

Thus, there may be a vicious circle among these factors, severe co-infection, multiple, persistent, and disseminated infections, AIGA titer, and CD4 + T cells level. This observation in our study is consistent with those of previous studies, including the study of Browne et al.^1^.

## Conclusions

This study provides the first evidence that high globulin and IgG levels, low CD4^+^T levels, double or multiple infections, and disseminated infections could be potentially effective predictive factors for AIGA positivity in HIV-negative patients with TM and/or NTM infections. Monitoring and predicting the AIGA titer is crucial to assess patient prognosis and host immunodeficiency severity. Methods for detecting AIGAs, including ELISA, particle-based assay, and flow cytometry, should be widely used in the clinical setting. A feasible, precise, and standardized protocol should be established to improve the diagnosis of AIGA in clinical practice.

This study had some limitations. First, this study was a multicenter retrospective study conducted in Guangxi, China, and its conclusions may not apply to other countries and provinces. The sample size was too small to perform multivariate analysis. However, this study makes a novel contribution to the literature by comparing AIGA-positive and AIGA-negative patients to elucidate the clinical significance of AIGAs and screening existing clinical indicators to identify potential predictors of AIGA for timely identification of AIGA positivity to evaluate host immunity and improve prognosis.

## Methods

We performed this multicenter prospective cohort study in Guangxi Province in the south of China between January 1, 2017, and December 31, 2019, from 13 centers [(1) The Eighth Affiliated Hospital of Sun Yat-Sen University; (2) The First Affiliated Hospital of Guangxi Medical University; (3) The Affiliated Tumor Hospital of Guangxi Medical University; (4) The Second Affiliated Hospital of Guangxi Medical University; (5) The Hospital of Guangxi Zhuang Autonomous Region; (6) Nan Xishan Hospital of Guangxi Zhuang Autonomous Region; (7) Nanning Second People's Hospital; (8) Nanning Fourth People's Hospital; (9) Nanning Eighth People's Hospital; (10) Yiyang Central Hospital; (11) Liuzhou First People's Hospital; (12) Guigang First People's Hospital; and (13) Guilin First People's Hospital].

We selected and collected peripheral blood serum from patients with fungal and bacterial infections (tuberculosis, NTM, TM, NTM, cryptococcus and other bacterial infections) during the study period. After detecting AIGA in the serum, we assigned patients into the AIGA positive group and AIGA negative group according to AIGA titers. Participants with AIGA titers exceeding the 99th percentile of the 103 healthy controls were defined as AIGA-positive. To eliminate selection bias, after matching the patients’ sex, age, HIV and the type of pathogenic microorganism infection, we assigned patients into the AIGA negative group from AIGA negative patients. We excluded participants less than 18 years of age; those with autoimmune disease, cancer, or immunodeficiency; or those who had received immunosuppressive medications in the past 3 months. Finally, we enrolled 63 AIGA-positive (group 1) and 29 AIGA-negative (group 2) patients with TM and NTM infections. Demographic and clinical data were recorded in standardized forms. The comparison of biomarkers or clinical parameters between the groups was performed at the active infection stage. All patients were followed-up until October 1, 2020, or until the time of death.

The clinical course of the infected patients was classified into the following four categories: cured (no recurrence of infection for at least 6 months after the discontinuation of antimicrobial therapy); persistent infection (no improvement of clinical symptoms after antimicrobial therapy); relapse infection (improvement of clinical symptoms, negative pathogen detection after antimicrobial therapy, followed by the reappearance of pathogen-associated infectious signs and/or positive pathogen testing); and death^15^. Disseminated disease was defined as an infection in two noncontiguous and sterile sites. Antimicrobial therapies included antifungal, anti-NTM, anti-tuberculosis, and anti-bacterium therapies.

This study was approved by the Ethical Review Committee of the First Affiliated Hospital of Guangxi Medical University (2020.KY-E-044). Written informed consent was provided by all participants enrolled in this study.

### Diagnostic criteria for NTM, TM, and other pathogenic infections

Each patient fulfilled the diagnostic criteria for each disease. TM infection was diagnosed as follows: 1) positive cultures of TM, characterized by dimorphic fungi that grew as a mold at 25 °C and yeast at 37 °C; 2) characteristic morphology of the yeast form of TM, confirmed by cytology and histopathology from tissues and secretions by Periodic Acid–Schiff staining or Wright’s staining, including a transverse septum^7,15^; or 3) TM isolated by metagenomic next-generation sequencing from clinical specimens. NTM was diagnosed following the 2007 American Thoracic Society (ATS)/Infectious Disease Society of America guidelines^16^. Positive cultures, cytology, and histopathology of the clinical specimens were used to diagnose *S. aureus*, *Aspergillus*, *Salmonella*, *Burkholderia*, as well as *Candida albicans*, *Klebsiella pneumoniae*, *Providencia rettgeri*, and *Citrobacter* infections.

### Healthy volunteer inclusion criteria

Healthy volunteers were enrolled over the same period. Healthy volunteers were defined as individuals without infection, underlying diseases, immunodeficiency conditions (diseases that could lead to or are associated with immunosuppression), including autoimmune diseases, malignancy, and primary immunodeficiency, or chronic diseases such as chronic renal failure, liver cirrhosis, diabetes mellitus, hypertension, or solid organ transplantation. Moreover, only those who had not received glucocorticoid and/or immunosuppressive therapy were included.

One hundred three healthy volunteers with normal routine blood and chest radiography findings were recruited from the health checkup center in The First Affiliated Hospital of Guangxi Medical University. All participants were HIV-negative.

### AIGA assay

Serum samples were obtained under sterile conditions before the patients received antimicrobial therapy and during the active stage of infection. Serum samples were retrieved from a serum bank and stored at − 80 °C. AIGAs were detected in all participants. All serum samples were tested at the first thaw. The detection of AIGAs was performed using an ELISA kit (Cloud-Clone Corp., Wuhan, China), whose detection range is 12–200 ng/ml. According to the manufacturer’s protocols, the serum samples from patients were 1:1500 diluted, and serum samples from a healthy control were 1:600 diluted by phosphate-buffered saline (PBS)^15^.

The normal range for the anti-IFN-γ-autoantibody concentration was defined by the 99^th^ percentile for the 103 healthy controls and was estimated using log-normal distribution. Outlying concentrations were classified as positive for anti-IFN-γ autoantibodies^1,6,15^.

### Statistical analysis

Data are expressed as median ± interquartile range. Differences between groups were compared using Kruskal–Wallis H or Mann–Whitney U test. Dunn–Bonferroni test was used for post-hoc analysis. Chi-square or Fisher’s exact test was used to compare categorical variables. Pearson correlation analysis was used for the correlation between AIGAs and clinical indices. Univariate logistic regression analysis was used for predicting factors of AIGA positivity. The normal range for the AIGA concentration was defined by the 99^th^ percentile for the 103 healthy controls and was estimated using log-normal distribution. Outlying concentrations were classified as positive for AIGAs^1,6^. We used SPSS (version 25.0) and GraphPad Prism (version 7, La Jolla, CA, USA) for statistical analysis and preparing graphs. Results with *P* < 0.05 were considered significant.

### Ethics approval and consent to participate

This study was approved by the Ethical Review Committee of the First Affiliated Hospital of Guangxi Medical University (2020.KY-E-044). Written informed consent was provided by all participants in the prospective cohort study. All patients were followed-up until July 1, 2020, or until the date of death. All methods were carried out in accordance with the Declaration of Helsinki guidelines and regulations.

## Data Availability

The datasets used or analyzed during the current study are available from the corresponding author on reasonable request.

## Abbreviations

BMI: Body mass index
AIGA: Anti-IFN-γ autoantibody
ND: Not done
HLA: Human leukocyte antigen
WBC: White blood cell
N: Neutrophil count
L: Lymphocyte count
HGB: Hemoglobin
ESR: Erythrocyte sedimentation rate
CRP: C-reactive protein
Ig: Immunoglobulin
